# Supplementary material for: A Humanized Yeast Phenomic Model of Deoxycytidine Kinase to Predict Genetic Buffering of Nucleoside Analog Cytotoxicity
Source: Genes (Basel). 2019 Sep 30;10(10):770. doi: 10.3390/genes10100770 (PMC6826991; doi:10.3390/genes10100770)
Supplement: Supplementary file 1 [file genes-10-00770-s001.zip › Additional_Files/Additional_File7_GOTermHeatmaps.zip!/Additional_File7_GOTermHeatmaps/ER_to_Golgi_vesicle-mediated_transport.pdf]

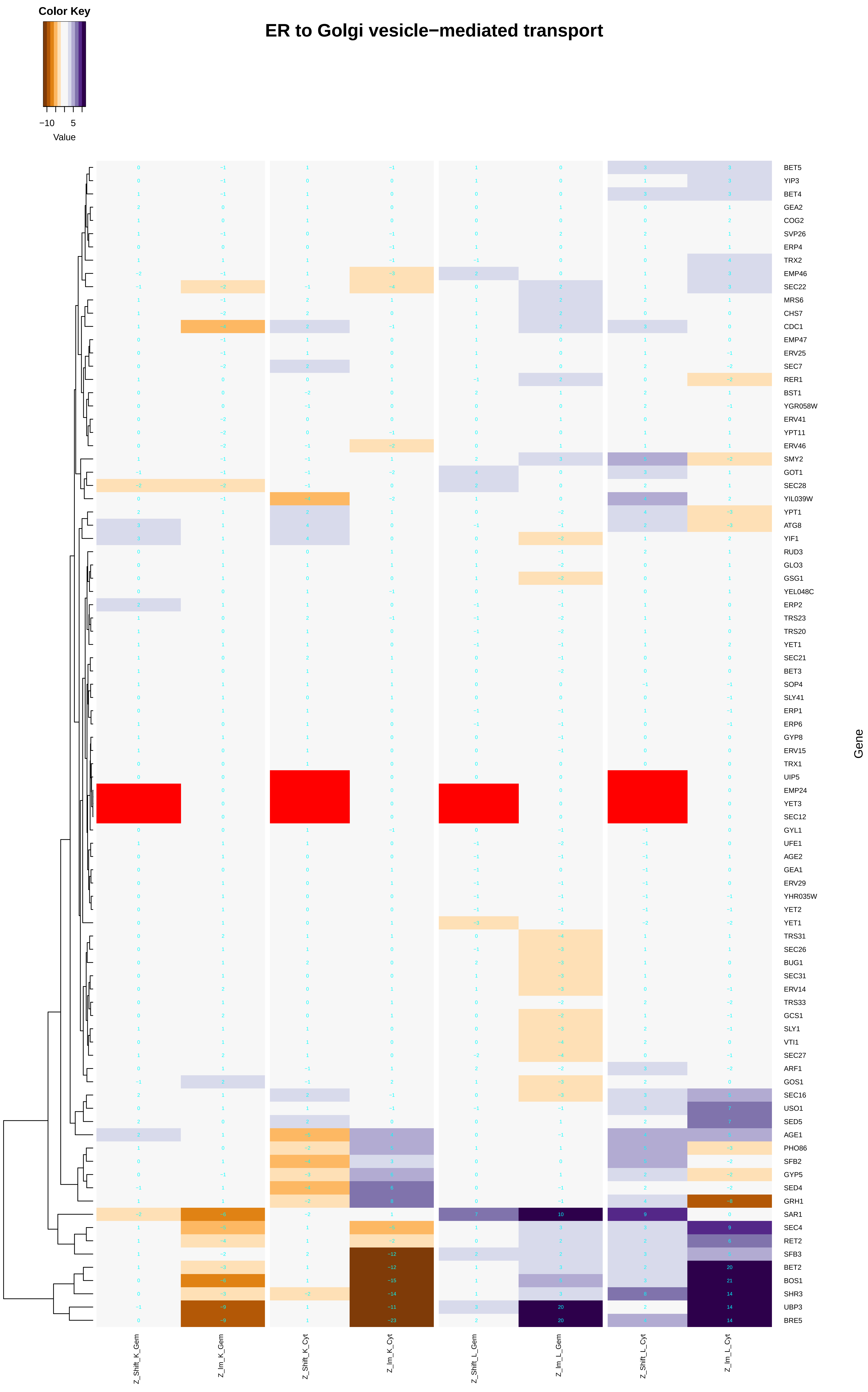
Color Key
−10
5
Value
ER to Golgi vesicle−mediated transport
BET5
YIP3
BET4
GEA2
COG2
SVP26
ERP4
TRX2
EMP46
SEC22
MRS6
CHS7
CDC1
EMP47
ERV25
SEC7
RER1
BST1
YGR058W
ERV41
YPT11
ERV46
SMY2
GOT1
SEC28
YIL039W
YPT1
ATG8
YIF1
RUD3
GLO3
GSG1
YEL048C
ERP2
TRS23
TRS20
YET1
SEC21
BET3
SOP4
SLY41
ERP1
ERP6
GYP8
ERV15
TRX1
UIP5
EMP24
YET3
SEC12
GYL1
UFE1
AGE2
GEA1
ERV29
YHR035W
YET2
YET1
TRS31
SEC26
BUG1
SEC31
ERV14
TRS33
GCS1
SLY1
VTI1
SEC27
ARF1
GOS1
SEC16
USO1
SED5
AGE1
PHO86
SFB2
GYP5
SED4
GRH1
SAR1
SEC4
RET2
SFB3
BET2
BOS1
SHR3
UBP3
BRE5
Gene
Z_Shift_K_Gem
Z_Im_K_Gem
Z_Shift_K_Cyt
Z_Im_K_Cyt
Z_Shift_L_Gem
Z_Im_L_Gem
Z_Shift_L_Cyt
Z_Im_L_Cyt
